# Diimidazolium phthalate monohydrate

**DOI:** 10.1107/S1600536812028619

**Published:** 2012-06-30

**Authors:** Chua-Hua Yu

**Affiliations:** a Ordered Matter Science Research Center, Southeast University, Nanjing 211189, People’s Republic of China

## Abstract

In the title compound, 2C_3_H_5_N_2_^+^·C_8_H_4_O_4_^2−^·H_2_O, the cations, anion and water mol­ecule are connected by N—H⋯O and O—H⋯O hydrogen bonds, forming a three-dimensional network.

## Related literature

The title compound was synthesized during a search for ferroelectric materials. For background to ferroelectric organic materials with framework structures, see: Zhang *et al.* (2009 ▶, 2010 ▶); Zhang & Xiong (2012 ▶). For related structures, see: Yu & Zhu (2012 ▶); Zhu & Yu (2011 ▶).
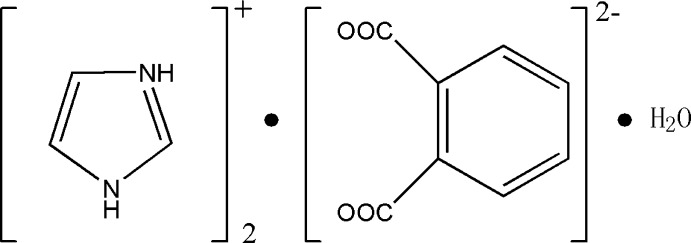


## Experimental

### 

#### Crystal data

2C_3_H_5_N_2_^+^·C_8_H_4_O_4_^2−^·H_2_O
*M* *_r_* = 320.31
Monoclinic, 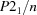

*a* = 9.1250 (18) Å
*b* = 12.979 (3) Å
*c* = 13.549 (3) Å
β = 103.85 (3)°
*V* = 1558.0 (6) Å^3^
*Z* = 4
Mo *K*α radiation
μ = 0.11 mm^−1^
*T* = 293 K
0.32 × 0.28 × 0.26 mm

#### Data collection

Rigaku SCXmini diffractometer
Absorption correction: multi-scan (*CrystalClear*; Rigaku, 2005 ▶) *T*_min_ = 0.967, *T*_max_ = 0.973
15753 measured reflections
3567 independent reflections
2758 reflections with *I* > 2σ(*I*)
*R*_int_ = 0.044
3 standard reflections every 180 reflections intensity decay: none

#### Refinement

*R*[*F* ^2^ > 2σ(*F* ^2^)] = 0.058
*wR*(*F* ^2^) = 0.141
*S* = 1.10
3567 reflections
208 parameters
H-atom parameters constrained
Δρ_max_ = 0.32 e Å^−3^
Δρ_min_ = −0.50 e Å^−3^

### 

Data collection: *CrystalClear* (Rigaku, 2005 ▶); cell refinement: *CrystalClear*; data reduction: *CrystalClear*; program(s) used to solve structure: *SHELXS97* (Sheldrick, 2008 ▶); program(s) used to refine structure: *SHELXL97* (Sheldrick, 2008 ▶); molecular graphics: *DIAMOND* (Brandenburg & Putz, 2005 ▶); software used to prepare material for publication: *SHELXL97*.

## Supplementary Material

Crystal structure: contains datablock(s) I, global. DOI: 10.1107/S1600536812028619/go2058sup1.cif

Structure factors: contains datablock(s) I. DOI: 10.1107/S1600536812028619/go2058Isup2.hkl

Supplementary material file. DOI: 10.1107/S1600536812028619/go2058Isup3.cml

Additional supplementary materials:  crystallographic information; 3D view; checkCIF report

## Figures and Tables

**Table 1:** Hydrogen-bond geometry (Å, °)

*D*—H⋯*A*	*D*—H	H⋯*A*	*D*⋯*A*	*D*—H⋯*A*
N1—H1*A*⋯O1	0.96	1.71	2.665 (2)	170
N2—H2*A*⋯O2^i^	0.91	1.75	2.655 (2)	172
N3—H3*A*⋯O3	0.95	1.80	2.747 (2)	177
N3—H3*A*⋯O4	0.95	2.56	3.191 (2)	124
N4—H4*A*⋯O3^ii^	0.86	1.93	2.774 (2)	168
O1*W*—H1*WA*⋯O4	0.86	1.98	2.834 (2)	170
O1*W*—H1*WB*⋯O2^iii^	0.90	1.94	2.8326 (19)	172

Symmetry codes: (i) 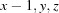; (ii) 
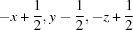
; (iii) 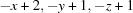.

**Fig. 1.**
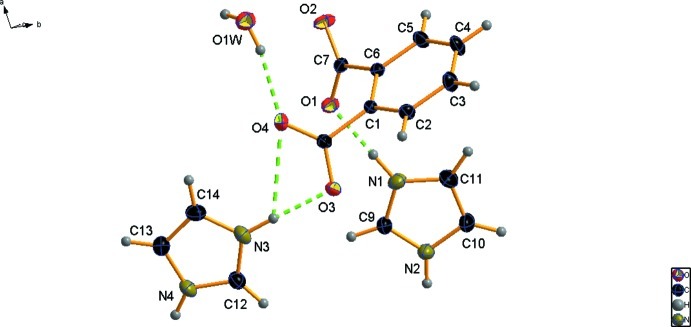
A diagram of the title compound, with displacement ellipsoids drawn at the 30% probability level. The atomic numbering scheme is shown.

**Fig. 2.**
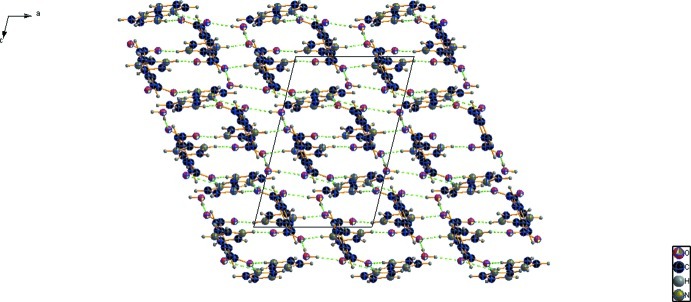
A view of the packing diagram of the title compound, stacking along the b axis. Hydrogen bonds are shown as dashed lines.

## supplementary crystallographic information

### Comment

In our search for potential ferroelectric phase change materials, the title compound was synthesized. This search is carried out by measurement of the dielectric constant of compounds on the basis of temperature, for eaxmple, (Zhang, Chen *et al.*, 2009; Zhang, Ye *et al.*, 2010; Zhang & Xiong, 2012), this has been carried out for C_3_H_5_N_2_^+^.C_2_HO_4_^-^ (Yu & Zhu, 2012) and C_5_H_9_N_2_^+^.C_8_H_5_O_4_^-^ (Zhu & Yu, 2011). In the case of the title compound no dielectric anomaly was observed ranging from 130 K to 375 K.

The crystal structure of the title compound contains two protonated imidazolium cations, one phthalate anion, losing two H atoms, and one molecule of water molecule is shown in Fig. 1. The asymmetric unit was selected with the cations, anion and water molecule connected by the intramolecular hydrogen bonds, N1–1A···O1, N3–H3A···O3, N3–H3A···O4 and O1W–H1WA···O4 all of which connect the cations and the water molecule to the anion, Table 1. These units are connected by the intermolecular hydrogen bonds, N2–H2A···O2(-1+x,y,z), N4-H4A···O3(-x+1/2,y-1/2,-z+1/2) and O1W–H1WB···O2(-x+2,-y+1,-z+1), to form a three-dimensional network Table2 and Figure 1.

### Experimental

0.83 g (5 mmol) of phthalic acid was added to in 10 ml water which was heated. A few drops of ethanol and 0.68 g (10 mmol) imidazole were added to the solution. The mixture was stirreduntil it reached ambient temperature, the liquid was filtered to give a clear solution. Colourless crystals suitable for X-ray structure analysis were obtained by the slow evaporation of the solution after several days at the ambient temperature.

### Refinement

H atoms attached to C were placed in calculated positions (C—H = 0.93 Å for *C_sp2_* atoms) while those attached to N and O were found in positions derived from a difference electron density map, with *U_iso_*(H) = 1.2 *U_iso_*(C, N), *U_iso_*(H) = 1.5 *U_iso_*(O). The -2 0 2 reflection was omitted since its measured value appear to be anomalous.

### Crystal data

**Table:**

2C_3_H_5_N_2_^+^·C_8_H_4_O_4_^2^^−^·H_2_O	*F*(000) = 672
*M**_r_* = 320.31	*D*_x_ = 1.366 Mg m^−^^3^
Monoclinic, *P*2_1_/*n*	Mo *K*α radiation, λ = 0.71073 Å
Hall symbol: -P 2yn	Cell parameters from 3567 reflections
*a* = 9.1250 (18) Å	θ = 3.1–27.5°
*b* = 12.979 (3) Å	µ = 0.11 mm^−^^1^
*c* = 13.549 (3) Å	*T* = 293 K
β = 103.85 (3)°	Block, colourless
*V* = 1558.0 (6) Å^3^	0.32 × 0.28 × 0.26 mm
*Z* = 4	

### Data collection

**Table:**

Rigaku SCXmini diffractometer	2758 reflections with *I* > 2σ(*I*)
Radiation source: fine-focus sealed tube	*R*_int_ = 0.044
Graphite monochromator	θ_max_ = 27.5°, θ_min_ = 3.1°
ω scans	*h* = −11→11
Absorption correction: multi-scan (*CrystalClear*; Rigaku, 2005)	*k* = −16→16
*T*_min_ = 0.967, *T*_max_ = 0.973	*l* = −17→17
15753 measured reflections	3 standard reflections every 180 reflections
3567 independent reflections	intensity decay: none

### Refinement

**Table:**

Refinement on *F*^2^	Primary atom site location: structure-invariant direct methods
Least-squares matrix: full	Secondary atom site location: difference Fourier map
*R*[*F*^2^ > 2σ(*F*^2^)] = 0.058	Hydrogen site location: inferred from neighbouring sites
*wR*(*F*^2^) = 0.141	H-atom parameters constrained
*S* = 1.10	*w* = 1/[σ^2^(*F*_o_^2^) + (0.0667*P*)^2^ + 0.3812*P*] where *P* = (*F*_o_^2^ + 2*F*_c_^2^)/3
3567 reflections	(Δ/σ)_max_ < 0.001
208 parameters	Δρ_max_ = 0.32 e Å^−^^3^
0 restraints	Δρ_min_ = −0.50 e Å^−^^3^

### Special details

**Table:**

Geometry. All e.s.d.'s (except the e.s.d. in the dihedral angle between two l.s. planes) are estimated using the full covariance matrix. The cell e.s.d.'s are taken into account individually in the estimation of e.s.d.'s in distances, angles and torsion angles; correlations between e.s.d.'s in cell parameters are only used when they are defined by crystal symmetry. An approximate (isotropic) treatment of cell e.s.d.'s is used for estimating e.s.d.'s involving l.s. planes.
Refinement. Refinement of *F*^2^ against ALL reflections. The weighted *R*-factor *wR* and goodness of fit *S* are based on *F*^2^, conventional *R*-factors *R* are based on *F*, with *F* set to zero for negative *F*^2^. The threshold expression of *F*^2^ > σ(*F*^2^) is used only for calculating *R*-factors(gt) *etc*. and is not relevant to the choice of reflections for refinement. *R*-factors based on *F*^2^ are statistically about twice as large as those based on *F*, and *R*- factors based on ALL data will be even larger.

### Fractional atomic coordinates and isotropic or equivalent isotropic displacement parameters (Å^2^)

**Table:**

	*x*	*y*	*z*	*U*_iso_*/*U*_eq_	
O1	0.67631 (14)	0.59458 (9)	0.53305 (10)	0.0375 (3)	
O2	0.92261 (14)	0.61977 (10)	0.57079 (11)	0.0423 (3)	
O3	0.45616 (13)	0.66464 (9)	0.28714 (10)	0.0386 (3)	
O4	0.67947 (15)	0.59102 (10)	0.30286 (12)	0.0466 (4)	
C1	0.66990 (17)	0.75577 (12)	0.37728 (12)	0.0242 (3)	
C2	0.64193 (19)	0.85209 (13)	0.33196 (13)	0.0310 (4)	
H2	0.5809	0.8575	0.2665	0.037*	
C3	0.7038 (2)	0.93997 (13)	0.38309 (14)	0.0385 (4)	
H3	0.6833	1.0041	0.3523	0.046*	
C4	0.7959 (2)	0.93247 (13)	0.47976 (15)	0.0417 (5)	
H4	0.8372	0.9915	0.5144	0.050*	
C5	0.8267 (2)	0.83681 (14)	0.52503 (13)	0.0353 (4)	
H5	0.8898	0.8319	0.5899	0.042*	
C6	0.76452 (17)	0.74796 (12)	0.47483 (12)	0.0254 (3)	
C7	0.79001 (18)	0.64612 (13)	0.52933 (12)	0.0266 (4)	
C8	0.59838 (19)	0.66201 (12)	0.31870 (12)	0.0275 (4)	
N1	0.41475 (17)	0.68481 (12)	0.53530 (12)	0.0390 (4)	
H1A	0.5080	0.6559	0.5268	0.047*	
N2	0.18863 (17)	0.69028 (12)	0.55394 (12)	0.0375 (4)	
H2A	0.0940	0.6703	0.5552	0.045*	
C9	0.2907 (2)	0.63160 (14)	0.52810 (15)	0.0385 (4)	
H9	0.2770	0.5631	0.5079	0.046*	
C10	0.2510 (2)	0.78435 (16)	0.58006 (18)	0.0499 (5)	
H10	0.2043	0.8406	0.6021	0.060*	
C11	0.3926 (2)	0.78088 (16)	0.5680 (2)	0.0548 (6)	
H11	0.4625	0.8344	0.5799	0.066*	
N3	0.37146 (18)	0.46491 (12)	0.23556 (11)	0.0375 (4)	
H3A	0.4030	0.5339	0.2521	0.045*	
N4	0.24322 (18)	0.32527 (12)	0.20991 (12)	0.0403 (4)	
H4A	0.1710	0.2820	0.2070	0.048*	
C12	0.2391 (2)	0.42411 (14)	0.23263 (15)	0.0398 (4)	
H12	0.1560	0.4590	0.2446	0.048*	
C13	0.3841 (2)	0.30217 (16)	0.19825 (16)	0.0446 (5)	
H13	0.4179	0.2383	0.1820	0.053*	
C14	0.4641 (2)	0.39014 (16)	0.21490 (16)	0.0452 (5)	
H14	0.5645	0.3983	0.2127	0.054*	
O1W	0.99551 (16)	0.57033 (11)	0.32839 (11)	0.0479 (4)	
H1WA	0.9020	0.5841	0.3250	0.072*	
H1WB	1.0210	0.5131	0.3660	0.072*	

### Atomic displacement parameters (Å^2^)

**Table:**

	*U*^11^	*U*^22^	*U*^33^	*U*^12^	*U*^13^	*U*^23^
O1	0.0274 (6)	0.0290 (6)	0.0568 (8)	−0.0006 (5)	0.0116 (6)	0.0092 (6)
O2	0.0250 (6)	0.0430 (8)	0.0569 (8)	0.0060 (6)	0.0058 (6)	0.0199 (6)
O3	0.0273 (7)	0.0295 (7)	0.0526 (8)	−0.0025 (5)	−0.0032 (6)	−0.0047 (6)
O4	0.0372 (8)	0.0359 (7)	0.0664 (9)	0.0010 (6)	0.0118 (7)	−0.0221 (6)
C1	0.0216 (7)	0.0219 (8)	0.0291 (8)	−0.0012 (6)	0.0060 (6)	−0.0019 (6)
C2	0.0316 (9)	0.0288 (9)	0.0295 (9)	0.0003 (7)	0.0015 (7)	0.0029 (7)
C3	0.0500 (12)	0.0205 (8)	0.0423 (10)	−0.0005 (8)	0.0057 (8)	0.0041 (7)
C4	0.0529 (12)	0.0236 (9)	0.0434 (11)	−0.0073 (8)	0.0016 (9)	−0.0066 (7)
C5	0.0380 (10)	0.0323 (9)	0.0307 (9)	−0.0044 (8)	−0.0013 (7)	−0.0039 (7)
C6	0.0219 (8)	0.0242 (8)	0.0299 (8)	0.0000 (6)	0.0056 (6)	0.0003 (6)
C7	0.0257 (8)	0.0252 (8)	0.0293 (8)	0.0029 (7)	0.0075 (6)	0.0009 (6)
C8	0.0265 (8)	0.0255 (8)	0.0288 (8)	−0.0020 (7)	0.0037 (6)	−0.0015 (6)
N1	0.0283 (8)	0.0405 (9)	0.0500 (9)	0.0023 (7)	0.0129 (7)	0.0026 (7)
N2	0.0290 (8)	0.0320 (8)	0.0522 (9)	−0.0025 (6)	0.0113 (7)	−0.0003 (7)
C9	0.0351 (10)	0.0295 (9)	0.0501 (11)	0.0001 (8)	0.0083 (8)	−0.0045 (8)
C10	0.0416 (12)	0.0293 (10)	0.0811 (16)	−0.0004 (9)	0.0194 (11)	−0.0093 (10)
C11	0.0416 (12)	0.0357 (11)	0.0872 (17)	−0.0119 (9)	0.0155 (11)	−0.0056 (11)
N3	0.0434 (9)	0.0313 (8)	0.0373 (8)	−0.0099 (7)	0.0088 (7)	−0.0018 (6)
N4	0.0394 (9)	0.0317 (8)	0.0490 (10)	−0.0117 (7)	0.0088 (7)	−0.0040 (7)
C12	0.0403 (11)	0.0346 (10)	0.0458 (11)	−0.0065 (8)	0.0126 (9)	−0.0049 (8)
C13	0.0446 (11)	0.0375 (10)	0.0508 (12)	0.0022 (9)	0.0100 (9)	−0.0068 (9)
C14	0.0340 (10)	0.0504 (12)	0.0527 (12)	−0.0067 (9)	0.0131 (9)	−0.0013 (10)
O1W	0.0393 (8)	0.0419 (8)	0.0624 (10)	0.0004 (6)	0.0116 (7)	0.0079 (7)

### Geometric parameters (Å, º)

**Table:**

O1—C7	1.246 (2)	N2—C9	1.314 (2)
O2—C7	1.253 (2)	N2—C10	1.358 (2)
O3—C8	1.266 (2)	N2—H2A	0.9055
O4—C8	1.232 (2)	C9—H9	0.9300
C1—C2	1.389 (2)	C10—C11	1.342 (3)
C1—C6	1.399 (2)	C10—H10	0.9300
C1—C8	1.513 (2)	C11—H11	0.9300
C2—C3	1.383 (2)	N3—C12	1.310 (2)
C2—H2	0.9300	N3—C14	1.360 (3)
C3—C4	1.380 (3)	N3—H3A	0.9507
C3—H3	0.9300	N4—C12	1.322 (2)
C4—C5	1.384 (3)	N4—C13	1.365 (3)
C4—H4	0.9300	N4—H4A	0.8591
C5—C6	1.389 (2)	C12—H12	0.9300
C5—H5	0.9300	C13—C14	1.345 (3)
C6—C7	1.505 (2)	C13—H13	0.9300
N1—C9	1.309 (2)	C14—H14	0.9300
N1—C11	1.355 (3)	O1W—H1WA	0.8623
N1—H1A	0.9615	O1W—H1WB	0.8997
			
C2—C1—C6	119.30 (14)	C9—N2—H2A	125.4
C2—C1—C8	118.78 (14)	C10—N2—H2A	126.5
C6—C1—C8	121.91 (14)	N1—C9—N2	109.25 (16)
C3—C2—C1	120.73 (15)	N1—C9—H9	125.4
C3—C2—H2	119.6	N2—C9—H9	125.4
C1—C2—H2	119.6	C11—C10—N2	107.09 (18)
C4—C3—C2	120.01 (16)	C11—C10—H10	126.5
C4—C3—H3	120.0	N2—C10—H10	126.5
C2—C3—H3	120.0	C10—C11—N1	107.11 (18)
C3—C4—C5	119.80 (16)	C10—C11—H11	126.4
C3—C4—H4	120.1	N1—C11—H11	126.4
C5—C4—H4	120.1	C12—N3—C14	108.62 (16)
C4—C5—C6	120.81 (16)	C12—N3—H3A	127.8
C4—C5—H5	119.6	C14—N3—H3A	123.5
C6—C5—H5	119.6	C12—N4—C13	108.66 (16)
C5—C6—C1	119.34 (15)	C12—N4—H4A	125.5
C5—C6—C7	119.47 (14)	C13—N4—H4A	125.7
C1—C6—C7	121.02 (14)	N3—C12—N4	108.79 (18)
O1—C7—O2	124.02 (15)	N3—C12—H12	125.6
O1—C7—C6	117.34 (14)	N4—C12—H12	125.6
O2—C7—C6	118.61 (14)	C14—C13—N4	106.43 (18)
O4—C8—O3	124.83 (15)	C14—C13—H13	126.8
O4—C8—C1	119.42 (15)	N4—C13—H13	126.8
O3—C8—C1	115.72 (14)	C13—C14—N3	107.50 (18)
C9—N1—C11	108.42 (17)	C13—C14—H14	126.2
C9—N1—H1A	124.0	N3—C14—H14	126.2
C11—N1—H1A	127.1	H1WA—O1W—H1WB	108.6
C9—N2—C10	108.12 (16)		
			
C6—C1—C2—C3	−1.4 (3)	C2—C1—C8—O4	122.83 (18)
C8—C1—C2—C3	179.38 (16)	C6—C1—C8—O4	−56.3 (2)
C1—C2—C3—C4	0.8 (3)	C2—C1—C8—O3	−55.5 (2)
C2—C3—C4—C5	0.3 (3)	C6—C1—C8—O3	125.37 (17)
C3—C4—C5—C6	−0.7 (3)	C11—N1—C9—N2	0.7 (2)
C4—C5—C6—C1	0.1 (3)	C10—N2—C9—N1	−0.9 (2)
C4—C5—C6—C7	−175.09 (17)	C9—N2—C10—C11	0.7 (3)
C2—C1—C6—C5	1.0 (2)	N2—C10—C11—N1	−0.3 (3)
C8—C1—C6—C5	−179.85 (16)	C9—N1—C11—C10	−0.2 (3)
C2—C1—C6—C7	176.10 (15)	C14—N3—C12—N4	−0.5 (2)
C8—C1—C6—C7	−4.7 (2)	C13—N4—C12—N3	0.2 (2)
C5—C6—C7—O1	125.27 (18)	C12—N4—C13—C14	0.1 (2)
C1—C6—C7—O1	−49.8 (2)	N4—C13—C14—N3	−0.4 (2)
C5—C6—C7—O2	−52.5 (2)	C12—N3—C14—C13	0.6 (2)
C1—C6—C7—O2	132.45 (17)		

### Hydrogen-bond geometry (Å, º)

**Table:**

*D*—H···*A*	*D*—H	H···*A*	*D*···*A*	*D*—H···*A*
N1—H1*A*···O1	0.96	1.71	2.665 (2)	170
N2—H2*A*···O2^i^	0.91	1.75	2.655 (2)	172
N3—H3*A*···O3	0.95	1.80	2.747 (2)	177
N3—H3*A*···O4	0.95	2.56	3.191 (2)	124
N4—H4*A*···O3^ii^	0.86	1.93	2.774 (2)	168
O1*W*—H1*WA*···O4	0.86	1.98	2.834 (2)	170
O1*W*—H1*WB*···O2^iii^	0.90	1.94	2.8326 (19)	172

Symmetry codes: (i) *x*−1, *y*, *z*; (ii) −*x*+1/2, *y*−1/2, −*z*+1/2; (iii) −*x*+2, −*y*+1, −*z*+1.

## References

[bb1] Brandenburg, K. & Putz, H. (2005). *DIAMOND* Crystal Impact GbR, Bonn, Germany.

[bb2] Rigaku (2005). *CrystalClear.* Rigaku Corporation, Tokyo, Japan.

[bb3] Sheldrick, G. M. (2008). *Acta Cryst.* A**64**, 112–122. 10.1107/S0108767307043930 18156677

[bb4] Yu, C.-H. & Zhu, R.-Q. (2012). *Acta Cryst.* E**68**, o1911. 10.1107/S1600536812023136 PMC3379467 22719665

[bb5] Zhang, W., Chen, L.-Z., Xiong, R.-G., Nakamura, T. & Huang, S.-P. (2009). *J. Am. Chem. Soc.* **131**, 12544–12545. 10.1021/ja905399x 19685869

[bb6] Zhang, W. & Xiong, R.-G. (2012). *Chem. Rev.* **112**, 1163–1195. 10.1021/cr200174w 21939288

[bb7] Zhang, W., Ye, H.-Y., Cai, H.-L., Ge, J.-Z., Xiong, R.-G. & Huang, S.-P. D. (2010). *J. Am. Chem. Soc.* **132**, 7300–7302. 10.1021/ja102573h 20459097

[bb8] Zhu, R.-Q. & Yu, C.-H. (2011). *Acta Cryst.* E**67**, o2746. 10.1107/S1600536811038578 PMC3201371 22058806

